# Dissecting the Variations of Ripening Progression and Flavonoid Metabolism in Grape Berries Grown under Double Cropping System

**DOI:** 10.3389/fpls.2017.01912

**Published:** 2017-11-10

**Authors:** Wei-Kai Chen, Xian-Jin Bai, Mu-Ming Cao, Guo Cheng, Xiong-Jun Cao, Rong-Rong Guo, Yu Wang, Lei He, Xiao-Hui Yang, Fei He, Chang-Qing Duan, Jun Wang

**Affiliations:** ^1^Center for Viticulture and Enology, College of Food Science and Nutritional Engineering, China Agricultural University, Beijing, China; ^2^Key Laboratory of Viticulture and Enology, Ministry of Agriculture, Beijing, China; ^3^Guangxi Academy of Agricultural Sciences, Nanning, China; ^4^Grape and Wine Research Institute, Guangxi Academy of Agricultural Sciences, Nanning, China; ^5^Guangxi Crop Genetic Improvement and Biotechnology Laboratory, Nanning, China

**Keywords:** transcriptome, secondary metabolism, subtropical climate, double cropping system, *Vitis vinifera*, flavonoid biosynthesis, ripening

## Abstract

A double cropping system has been commercially adopted in southern China, where there is abundant sunshine and heat resources. In this viticulture system, the first growing season normally starts as a summer cropping cycle; then, the vine is pruned and forced, resulting in a second crop in winter. Due to climate differences between the summer and winter growing seasons, grape ripening progression and flavonoid metabolism vary greatly. Here, the metabolites and transcriptome of flavonoid pathways were analyzed in grapes grown under two growing seasons at different stages. Notably, the winter cropping cycle strongly increased flavonoid levels by several times in comparison to summer grapes, while the summer season took a major toll on anthocyanin and flavonol accumulation, since the winter cropping greatly triggered the expression of upstream genes in the flavonoid pathway in a coordinated expression pattern. Moreover, the ratio of *VviF3′5′Hs* (flavonoid 3′5′-hydroxylase) to *VviF3*′*Hs* (flavonoid 3′-hydroxylase) transcript levels correlated remarkably well with the ratio of 3′5′-substituted to 3′-substituted flavonoids, which was presumed to control the flux of intermediates into different flavonoid branches. On the other hand, the phenological phase also varied greatly in the two crops. Compared to summer cropping, winter growing season accelerated the duration from budburst to veraison, therefore advancing the onset of ripening, but also prolonging the duration of ripening progression due to the purposes to harvest high-quality grapes. The differential expression pattern of hormone-related genes between the two cropping cycles might explain this phenomenon.

## Introduction

The growing season of grapevine is the time of year during which local climatic conditions (i.e., temperature, sunlight, and rainfall) permit its normal growth. In most temperate regions, grapevines undergo dormancy from late fall to early spring, and a single pruning and harvest is the conventional grapevine practice. However, in southern China, which shows extremely high temperature and concentrated rainfall during July and August, this traditional cultivation pattern does not adapt to local climate, and it takes a major toll on crop and grape quality, since these rainy months increase the occurrence of fungal diseases (Bai et al., 2008). In conventional practices, the compound buds remain in a stationary state during the current growing season, and they break dormancy in the following spring (Lavee and May, 1997). However, if these compound buds are forced out of dormancy early during the current season, a double cropping viticulture system can be achieved (Lin et al., 1985; Bai et al., 2008; Gu et al., 2012). Two crops of table grapes per year were achieved in Taiwan by a combination of pruning, defoliation, and chemical treatments (Lin et al., 1985). Similarly, in the Brazilian Southeast, the making of good wines was also attained by a double pruning approach (Favero et al., 2011). Therefore, the farmers in southern China introduced this strategy to overcome all detrimental environment under conventional practices.

**FIGURE 1:**
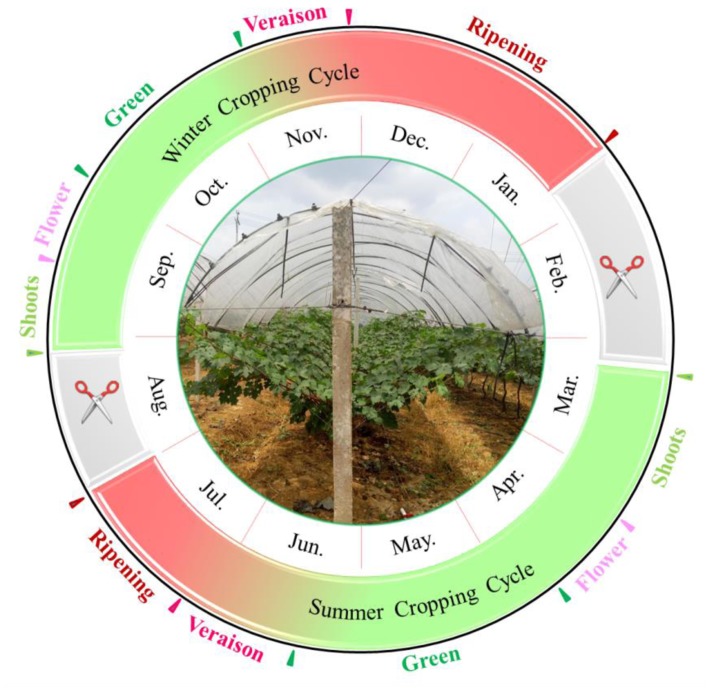
The schematic diagram of double cropping viticulture system.

With the benefit of high total yield and good quality, the double cropping system has great potential in subtropical viticulture regions (Lin et al., 1985). Recently, the winter grapes in Brazil showed physicochemical characteristics more suitable than those from the summer growing season for winemaking purposes, since the summer grapes were featured by higher cluster weight and titratable acidity while the winter crops were characterized by higher total soluble solids (TSS) content and pH value (Júnior et al., 2017). This viticulture system (**Figure 1**) has been commercially adopted in southern China, in which the first growing season normally starts in March to July; then, the vine is pruned and forced by a cyanamide solution in August, resulting in a second bud break in August, and a second crop until January of the following year (Lin et al., 1985; Bai et al., 2008). In some vineyards, the purpose of the first cropping is not to harvest grapes in summer, but to have the inflorescence primordia differentiated into latent buds (Dias et al., 2017). The second cropping resembles the extending of fruit ripening from summer to the autumn-winter of the growing season (Gu et al., 2012). This double cropping system could minimize the negative impact of a rainy and hot climate with the help of rain-shelter treatment, and it could maximize the use of sunshine and heat resources in subtropical regions.

Flavonoids are a group of natural compounds that share a common polyphenolic flavan skeleton. The biosynthetic pathways for anthocyanins, flavonols, and flavan-3-ols in plants share a common upstream step through phenylalanine ammonia-lyase (PAL) to flavanone 3-hydroxylase (F3H). Flavonol synthase (FLS) catalyzes dihydroflavonols to form their corresponding flavonol aglycones, and leads the flavonoid flux into the flavonol branch. On the other hand, leucoanthocyanidins and anthocyanidins can be converted into flavan-3-ols by leucoanthocyanidin reductase (LAR) and anthocyanidin reductase (ANR), respectively. As for anthocyanin synthesis, UDP-glucose: flavonoid 3-*O*-glucosyltransferase (UFGT) is considered as the key enzyme that determines the formation of anthocyanidin-3-*O*-glucosides. Due to internal and external differences between grapes, the flux through the flavonoid pathway toward downstream anthocyanins, flavonols, and flavan-3-ols varies significantly (Castellarin et al., 2007; Mouradov and Spangenberg, 2014). The climate of Nanning County in southern China is characterized by long growing seasons with higher temperature months from June to September. Many studies have shown that the flavonoid compounds of grape berries are greatly influenced by climate conditions (reviewed by Downey et al., 2006). High temperature often increases the degradation of flavonoid compounds, resulting in poor coloration (Yamane et al., 2006; Mori et al., 2007; Gu et al., 2012). Due to climate differences between the summer and winter growing seasons, especially the reverse temperature evolution pattern during grape berry development, flavonoid composition and content are often distinct between the two crops (Xu et al., 2011; Zhu et al., 2017). It is understandable that these variances in metabolites have much to do with the transcriptomic difference of grapes between the two growing seasons.

Since the first report of the draft genome sequence of the Pinot Noir grapevine (Jaillon et al., 2007), genome-wide transcriptome research revealing the regulation of berry ripening and its associated metabolic processes has become a hot topic (Fabres et al., 2017; Wong and Matus, 2017). Transcriptomic analyses also provide a comprehensive approach to study the transcriptional responses of grapes to changing environment conditions (Sun et al., 2017). Water deficit activates the expression of phenylpropanoid pathway transcripts, which increases flavonoid content in wine grape (Grimplet et al., 2007; Deluc et al., 2009; Savoi et al., 2016). Solar ultraviolet radiation triggers regulatory responses through the UV-B radiation-specific signaling pathway, which also results in the activation of phenylpropanoid biosynthesis (Carbonell-Bejerano et al., 2014). The transcriptional expression patterns of flavonoid biosynthesis in wine grapes grown in two regions with distinct climates also showed regional differences (Li et al., 2014). Furthermore, several mRNA expression profiling studies have been reported that show a detailed analysis of gene expression during grape berry development (Deluc et al., 2007, 2009; Sweetman et al., 2012; Degu et al., 2014). However, to the best of our knowledge, the transcriptomic analysis of seasonal variation on grape flavonoid compounds has not been reported within a double cropping system.

Thus, in the present study, we performed transcriptome and metabolite analysis of flavonoid biosynthesis both in summer- and winter-grapes of *Vitis vinifera* L. cv. “Cabernet Sauvignon” (CS) and *V. vinifera* cv. “Riesling” by RNA-sequencing and HPLC-ESI-MS/MS. Comparative analysis of the transcript and metabolite profiles revealed season- or cultivar-specific patterns of flavonoid biosynthesis. This paper provides insights into the mechanisms of growing season influence on flavonoid accumulation.

## Materials and Methods

### Experimental Vineyard and Double Cropping Viticulture Practices

The experiment was conducted in 2014 and 2015 on 7-year-old grapevines grafted onto SO4 rootstock in the vineyards of the Guangxi Academy of Agricultural Sciences, located in southern China (22°36′64″N, 108°14′13″E, altitude 104 m), where it is typically a subtropical humid monsoon climate with abundant sunshine and heat resources. The vines were planted in east-west rows under a rain-shelter treatment with an inter- and intra-row vine spacing of 3.5 m × 1.5 m and were managed in a closing Y-shaped training system with 2 × 4/5 shoots per meter and 1.0 m cordon above ground. Two widely planted wine grape cultivars, “CS” and “Riesling,” were selected for research.

The grape viticulture regime in the subtropical region is dominated by double cropping systems (Lin et al., 1985; Bai et al., 2008). In these cultivation practices, the vines are pruned twice and the grapes are harvested twice per year. To produce the first crop, the vines were pruned and enforced with 2.5–3.0% hydrogen cyanamide in mid-February when the temperature was maintained above 10°C. The terminal bud was not sprayed to avoid apical dominance. In addition, the soil was kept moist to accelerate germination. Then, the grapevines were in full bloom around mid-April, while the veraison stage ranged from mid-June to early July, followed by the harvest stage (summer grape). This whole period was termed the summer cropping cycle. Then, the vines were pruned and forced again in August, leading to the second crop (winter grape) in early January of the following year. In detail, the grapevines were pruned on approximately 20th August, were manually defoliated, and an average of 5–10 buds were left for each cane. Hydrogen cyanamide was smeared only at the terminal bud and in the vicinity of the pruning-wound surface. Then, the bud burst 5–8 days later and initiated the winter cropping cycle.

Grape berries in three biological replicates were collected at four E-L stages (Coombe, 1995) according to berry color and TSS (°Brix) for each crop as follows: pea-size berries (E-L 31), the onset of veraison (E-L 35), the end of veraison (E-L 36), and the harvest stage (E-L 38). In brief, 300 berries were selected from both the sunny and shady sides of at least 50 whole vine selections, among which 100-berry sub-samples were processed immediately to determine the physicochemical parameters. The remaining samples were frozen in liquid nitrogen and stored at -80°C for subsequent RNA and flavonoid extraction. TSS of the juices was determined with digital pocket handheld refractometer (PAL-1, Atago, Japan), and titratable acidity was measured by acid–base titration.

### Extraction of Flavonoid Compounds

The fresh skins were peeled from the grapes and were immediately ground into powder in liquid nitrogen. Afterward, the skin powder was lyophilized at -50°C and used for extraction of anthocyanin, flavan-3-ol, and flavonol. The anthocyanins and flavonols were simultaneously extracted in two analytical replicates according to a previous report (Downey et al., 2007). Three aliquots of grape skin powder (0.10 g) were immersed in 1.0 ml of 50% methanol in water, were ultrasound sonicated for 20 min, and were centrifuged. Then, the supernatant was collected and the residues were re-extracted again. All the supernatants were mixed, filtered through a 0.22-μm nylon membrane, and transferred to HPLC auto-sampler vials. The extraction of flavan-3-ol was also conducted twice for each sample according to method described by Liang et al. (2012). Grape skin powder (0.10 g) was mixed with 1 ml of phloroglucinol buffer (0.5% ascorbate, 300 mM HCl, and 50 g/l phloroglucinol in methanol), incubated at 50°C for 20 min, neutralized with 1 ml of sodium acetate (200 mM, pH 7.5), and finally centrifuged at 8000 × *g* for 15 min. This procedure was repeated three times, and the supernatants were combined and filtered for HPLC analysis.

### Analysis of Flavonoid Compounds

Analysis of flavonoid compounds was carried out using an Agilent 1200 Series HPLC–MSD trap VL equipped with a variable wavelength detector (for flavonol) or a diode array detector (for flavan-3-ol and anthocyanin). The mass spectrometric acquisition parameters were as follows: ESI interface, positive ion mode (for anthocyanin) or negative ion mode (for flavonol and flavan-3-ol), 35 psi nebulizer pressure, 10 ml/min drying N_2_ flow rate, 350°C drying N_2_ temperature, capillary voltage 3000 V, and scans at *m/z* 100–1000.

Anthocyanin extract was injected onto a Kromasil C18 column (250 mm × 4.6 mm, 5 μm). The mobile phases A and B were aqueous 2% formic acid and acetonitrile containing 2% formic acid, respectively. The flow rate was 1.0 ml/min, and the solvent gradients were as follows: from 6 to 10% B over 4 min, from 10 to 25% B over 8 min, isocratic 25% B for 1 min, from 25 to 40% B over 7 min, from 40 to 60% B over 15 min, from 60 to 100% B over 5 min, and from 100 to 6% B over 5 min. Other conditions were as follows: injection volume, 30 μl; detection wavelength, 525 nm; and column temperature, 50°C.

Flavan-3-ol compounds were separated on a reversed phase Zorbax SB-C18 column (250 mm × 4.6 mm, 5 μm) using mobile phase A of aqueous 0.2% acetic acid and mobile phase B of acetonitrile: 0.2% acetic acid (4:1) at a flow rate of 1 ml/min and were monitored at 280 nm at 25°C. The elution gradients of solvent B were as follows: 0 min, 10% B; 20 min, 10% B; 30 min, 15% B; 40 min, 20% B; 50 min, 33% B; 55 min, 40% B; 58 min, 100% B; 63 min, 100% B; and 64 min, 10% B.

For flavonol separation, mobile phase A was a mixture of formic acid:acetonitrile:water (85:50:865) and B was formic acid:acetonitrile:water:methanol (85:250:215:450). The column selected was a Zorbax SB-C18 (4.6 mm × 250 mm, 5 μm), with the temperature maintained at 40°C. The gradient conditions were as follows: 0% B over 7 min; 24.2 min, 14.2% B; 27 min, 15.7% B; 39 min, 23.5% B; 45 min, 26% B; 51.6 min, 32% B; 61.8 min, 40% B; 62.3 min, 60% B; 67.8 min, 100% B; and 78.4 min, 0% B. The flow rate was 0.63 ml/min, and the detector wavelength was 360 nm.

### Transcriptome Sequencing and Data Analysis

Three biological replicates for each sample were performed. A sub-sample of 50 berries were randomly selected from each biological replicate for RNA extraction. Total RNA was extracted from the frozen deseeded berries (whole pericarp) at three development stages (E-L 35, 36, and 38) using a Spectrum^TM^ Plant Total RNA Kit (Sigma–Aldrich, Carlsbad, CA, United States) to conduct transcriptome analysis on the Illumina HiSeq^TM^ 2000 platform with 50-bp single reads and were then aligned against the reference grapevine genome 12×V2, allowing no more than two mismatches. Transcriptome *de novo* assembly was conducted using the short reads assembling program Trinity with a fixed *k*-mer length of 25. To determine gene expression levels, the longest transcript was chosen to calculate the fragments per kilobases per million reads (FPKM) value when more than one transcript was obtained for a single gene (Mortazavi et al., 2008). For the functional annotation, unigene sequences were aligned to databases as described previously (Li et al., 2014). Differentially expressed genes (DEGs) between the samples were identified by the R package called “DESeq2.” A false discovery rate ≤0.01 and a fold change ≥2 were set as the threshold to judge the significance of gene expression differences. Gene Ontology (GO) and KEGG enrichment analysis of DEGs was used to select candidate genes. The data have been deposited in the NCBI Gene Expression Omnibus (GEO) database and are accessible through GEO accession GSE103226.

### Statistical Analysis

Heatmap visualizations were performed using the R package “pheatmap.” Principal component (PC) analysis was done using MetaboAnalyst 3.0^1^. A one-way analysis of variance (ANOVA) was used to measure the differences between flavonoid contents employing Duncan’s multiple range tests at *p* < 0.05. The column plots were prepared using OriginPro 9.2 (OriginLab Corporation, Northampton, MA, United States).

## Results

### Meteorological Data and Phenological Characteristics

**FIGURE 2:**
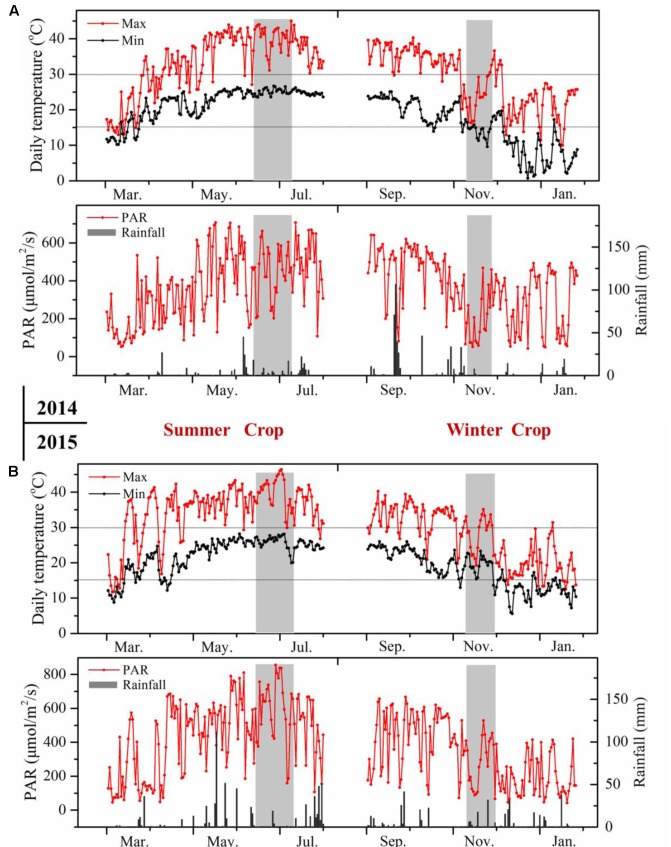
Meteorological data of the vineyards during grape berry development in 2014 **(A)** and 2015 **(B)**. The pink rectangle indicates veraison stage from E-L 35 to E-L 36.

The environmental condition in southern China corresponds to a typically subtropical humid monsoon climate, which is characterized by a hot and humid summer and a mild to cool winter (Supplementary Table S1). The climatic conditions displayed great differences between the two growing seasons (**Figure 2**). Basically, the daily temperature showed reverse evolution patterns in the two growing seasons from budbreak to harvest. In the summer cropping cycle, the temperature was low at an early stage, then it increased gradually until the veraison and ripening stages. The mean temperature in the summer season approached 30°C, and the daily maximum temperature frequently exceeded 35°C, which has been considered as detrimental to plant growth and anthocyanin accumulation (Cohen et al., 2012; de Rosas et al., 2017). While in the second cropping cycle, the temperature dropped from the point of flowering until the ripening process, with a mean temperature of approximately 20°C during the entire winter cropping. In addition, the extremely high temperature hours in the winter season during grape berry ripening was almost negligible, only 2 h versus 210 h in the summer. Another important difference in climate was rainfall. Southern China always experiences abundant and concentrated rainfall in the summer or, to a lesser extent, in the autumn. The heavy rainfall in June and July could greatly reduce grape quality, making the double cropping practice appealing, as this approach harvested double crops and shifted fruit ripening from the hot and rainy summer (July and August) to the mild and cool winter (January) in each growing season (Bai et al., 2008). However, the sunshine hours during berry development, ranging from fruit-set to harvest, were distinctly higher in summer cropping in both years, and this was also the case for the growing degrees days and photosynthetically active radiation. Diurnal temperature between day and night was another important climatic index (Cohen et al., 2012), and it was 1–4°C lower in winter cropping.

Due to climate difference in the two growing seasons, the phenological phase also varied greatly in the two crops (Supplementary Table S1). The entire period of berry development in winter cropping, from fruit-set to full-ripeness, was longer than or equal to that of the summer cycle, but the duration of the early phenological phase was hastened and the late progression was delayed. The winter cropping was characterized by shorter green and veraison stages, but a longer berry ripening stage. Compared to stage durations of cv. “CS” described in single cropped vineyards (Li et al., 2014), both crops showed shorter berry development periods partially due to higher temperature. The grapes of summer crop showed a longer veraison stage than that from single cropped vineyards, which was nearly equal to that in winter season. Comparing the ripening duration, the single cropped grapes ranged between the summer crop and winter crop herein (Li et al., 2014). With regard to whole duration from flowering to harvest, the years of 2014 and 2015 showed opposite results. The difference on the ripening duration in the winter cropping was behind this phenomenon, resulting from distinct differences in rainfall. It was noted that “CS” and “Riesling” demonstrated similar flowering, veraison, and harvest dates under the double cropping system.

**FIGURE 3:**
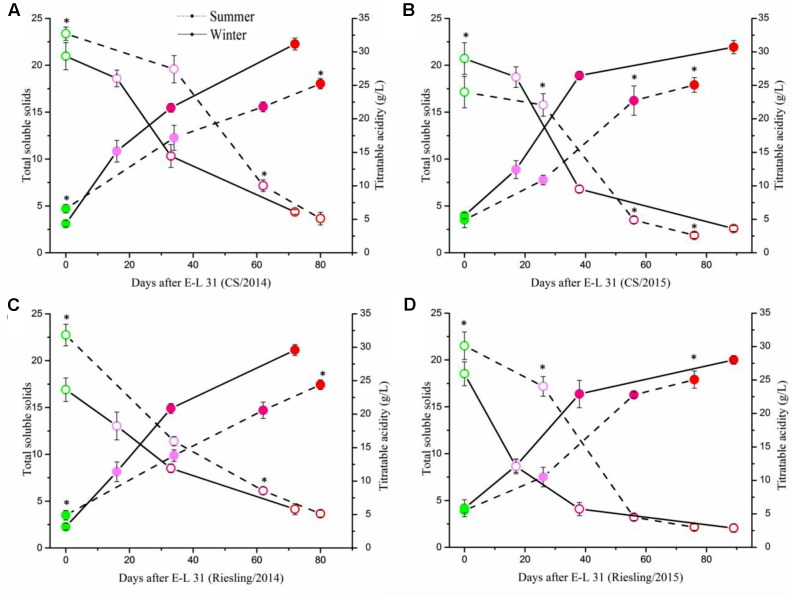
Evolution of total soluble solids and titratable acidity in ‘Cabernet Sauvignon’ **(A,B)** and ‘Riesling’ **(C,D)** grapes grown under double cropping system in 2014 **(A,C)** and 2015 **(B,D)**. The four points from green to red referred to stage of E-L 31/35/36/38, respectively. The asterisk indicates significant differences between the samples from the same stage.

To determine the impact of the cropping cycles on berry development and flavonoid metabolism, we collected berries at four development stages for each crop. The berries in summer cropping showed higher TSS at E-L 31, but showed an opposite result at E-L 38, and no significant difference was found at E-L 35 and E-L 36 (**Figure 3**). Berries sampled from both crops in both cultivars showed similar patterns of physicochemical characteristics during grape berry development (**Supplementary Figure S1**). Berry weight increased along with berry development, but the berries in the winter cropping were significantly smaller than those of the summer cropping at E-L 35, 36, and 38. The same phenomenon also occurred in berries from two separate years, with smaller berries in 2014 than that in 2015, which coincided with rainfall.

### Impact of Cropping Season on Flavonoid Metabolites across Berry Development

**FIGURE 4:**
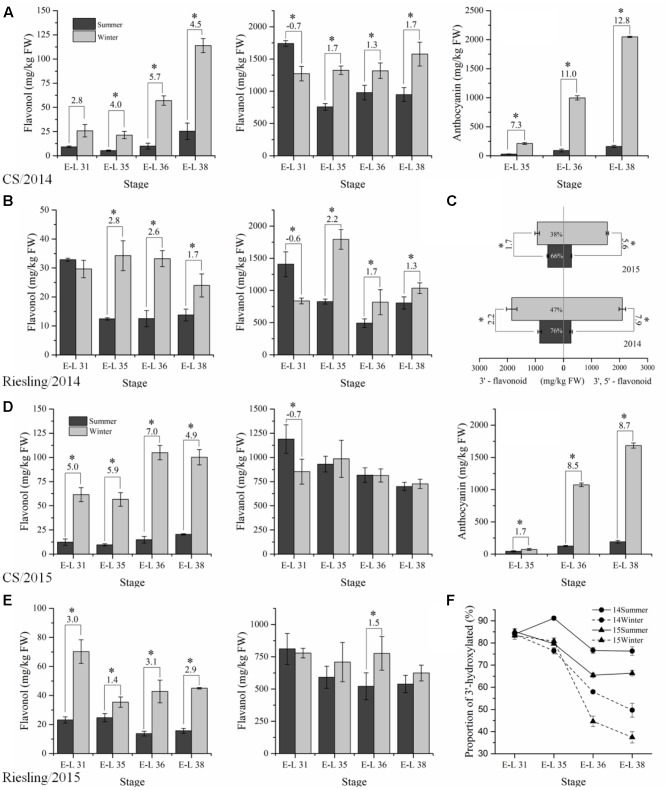
The composition and content of flavonoid compounds extracted in “CS” **(A,D)** and “Riesling” **(B,E)** skins under double cropping viticulture system in 2014 **(A,B)** and 2015 **(D,E)**, the profiles of flavonoid at harvest stage in “CS” **(C)** and the proportion of 3′4′-hydroxylated flavonoid in “CS” **(F)**.

The flavonoid compounds were analyzed to explore the effect of the double cropping system on grape berries in a subtropical region and to compare the metabolites with the transcriptome. A total of 14 and 8 flavonol glycosides were identified in “CS” and “Riesling,” respectively, which corresponded to six types of free aglycone in “CS” and three types in “Riesling” (Supplementary Table S2). The content of total flavonols in both “CS” and “Riesling” was significantly higher in winter grapes than the content in summer grapes, except for a single point of E-L 31 of “Riesling” in 2015 (**Figure 4**), in agreement with the data reported by Zhu et al. (2017). The total flavonol content showed an increasing trend for “CS,” but a declining trend for “Riesling.” It has been suggested that kaempferol-type, quercetin-type, and isorhamnetin-type flavonols are generally present in both red and white grapes, while myricetin-type flavonol and its derivatives are only accounted for in *V. vinifera* red grapes, which is in agreement with the present study (Castillo-Muñoz et al., 2007, 2010). For “CS” grapes, quercetin and myricetin were the most abundant flavonols, the proportion of which showed an inverse relationship during berry development. Quercetin accounted for more than 90% of the flavonol at stage E-L 31, while at E-L 38, its proportion reduced to 60–70% in summer grapes and 40–50% in winter grapes. Similarly, the proportion of glucoside and glucuronide also varied with the development stage and growing season. The green stage featured 3-*O*-glucuronides, while the harvest stage was characterized by 3-*O*-glucosides. In addition, the proportion of 3-*O*-glucosides at E-L 38 was 10% lower in winter grapes than in summer grapes. However, in “Riesling” grapes, the flavonol content was dominated by quercetin and 3-*O*-glucuronide, and their proportion showed no consistent trend versus the developmental stage.

The anthocyanins in “CS” displayed progressive accumulation in both crops across grape development. The content of total anthocyanins at E-L 38 in winter grapes, which was 2047.4 mg/kg FW in 2014 and 1682.3 mg/kg FW in 2015, was almost 10-fold higher than the content in summer grapes (**Figure 4**). In addition, the individual accumulation patterns of delphinidin-3-*O*-glucoside, cyanidin-3-*O*-glucoside, petunidin-3-*O*-glucoside, peonidin-3-*O*-glucoside, and malvidin-3-*O*-glucoside, and their acylated derivatives all showed similar trends (Supplementary Table S2). Malvidin-3-*O*-glucoside and its acylated derivatives accounted for 80% of the anthocyanins in summer grapes, while in winter grapes, the proportion of malvidin was significantly lower, dropping to 45% in 2014 and 60–70% in 2015. In addition, there was an increase in the percentages of delphinidin and petunidin in winter grapes in both years, but an increase in the proportion of cyanidin and peonidin occurred only in 2014. Thus, the content of 3′5′-substituted anthocyanins increased drastically in winter grapes relative to summer grapes, but its proportion sometimes decreased. Additionally, the ratios of methylated/non-methylated and acylated/non-acylated anthocyanins also showed significant differences between the two growing seasons, with higher methylation and acylation levels in summer grapes. In fact, the proportion of acylated anthocyanins was previously shown to greatly decrease with exposure to sunlight and high temperatures (Mori et al., 2007; Tarara et al., 2008).

Flavan-3-ols, the immediate competitors of the precursors for flavonol and anthocyanin synthesis, showed a mild reduction in total content as the stage progressed in the summer crop and maintained a relatively stable level in winter grapes, with the exception of “Riesling” in the 2015 winter crop, which displayed a transient peak at E-L 35, indicating the relatively limited effects of the cropping cycle on flavan-3-ols (**Figure 4**). The content of flavan-3-ols was higher in “CS” berries than “Riesling” berries, as well as flavonol. Comparing the total flavan-3-ol content between the two cropping cycles, the result in 2014 showed significant differences, but no significant difference was found in 2015. It seemed that the summer grapes had more flavanol at E-L 31, after which were reduced to lower levels than that in winter crops at later stages. Zhu et al. (2017) also found higher flavan-3-ol levels in winter grapes of cv. “Muscat Hamburg” at maturity, but no consistent trend was found in cv. “Khoyo.” In regard to flavanol profiles, epicatechin, as expected, was the most abundant fraction in both varieties, and its proportion was slightly but significantly higher in winter grapes at E-L 38. Epicatechin-3-*O*-gallate was the second main constituent, and it accounted for approximately 20% of the total composition, but showed no detectable differences between the two growing seasons.

Dihydrokaempferol represents the branching node in the flavonoid pathway, and it converts to dihydroquercetin and dihydromyricetin with the catalysis of F3′H (flavonoid 3′-hydroxylase) and F3′5′H (flavonoid 3′5′-hydroxylase), respectively, giving rise to the 3′-substituted and 3′5′-substituted flavonoid compounds (Degu et al., 2014). Since almost no 3′5′-substituted flavonoids existed in “Riesling” grapes, we only analyzed the proportion of 3′-hydroxylated and 3′5′-hydroxylated flavonoids in “CS” (**Figure 4**). At E-L 31, there was no significant difference among the four groups in the proportion of 3′-substituted flavonoids (approximately 85%). Then, their proportion gradually decreased and displayed significant differences at E-L 38, approaching 65–80% in summer grapes and 35–50% in winter grapes at E-L 38, due to an abundant accumulation of 3′5′-substituted anthocyanins and flavonols.

### Transcriptomic Changes of Flavonoid Pathway under Double Cropping System

**FIGURE 5:**
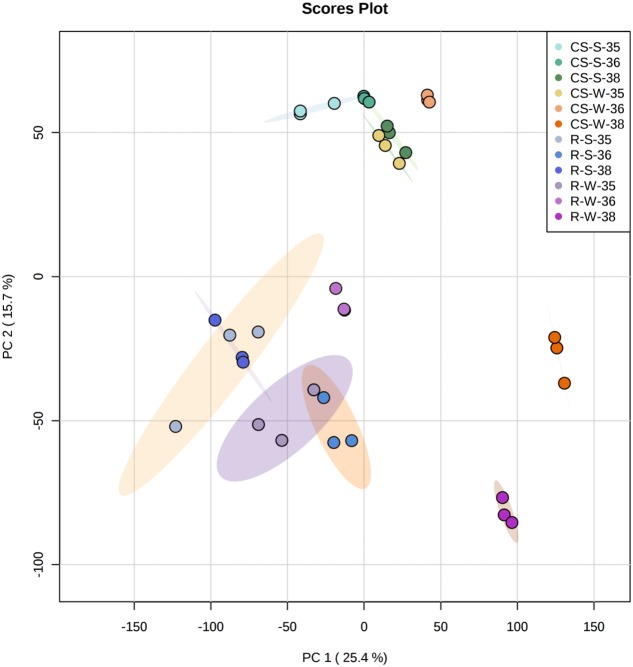
Principal component analysis of the whole normalized gene expression dataset. Four different colors (one per each type of sample: CS-W, CS-S, R-W, R-S) and then for each color three different tones (35, 36, and 38 EL stages) were used to represent different samples. Ellipses encircle the three replicates of each sample subjected to the same stage. CS/R, “Cabernet Sauvignon”/“Riesling”; S/W, summer/winter crop.

**FIGURE 6:**
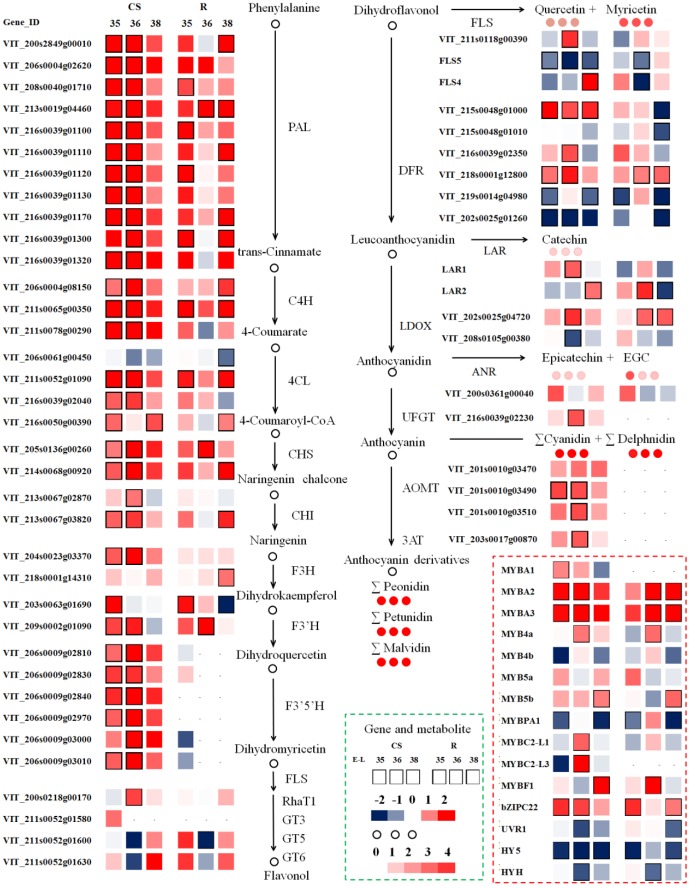
Modulation in berry transcripts involved in the phenylpropanoid and flavonoid pathway in summer and winter berries in 2014. Blue and red boxes indicate downregulated and upregulated transcripts, respectively, in winter berries versus large berries. Boxes with bold margins indicate differential expressed genes between two cropping cycles at a given developmental stage. Circles represent metabolites and their colors represent the fold changes in metabolite in “CS” at a given ripening stage when comparing winter berries versus control summer berries. TFs (lower right corner) involved in the regulation of the phenylpropanoid and the flavonoid pathway transcripts are depicted in dotted red rectangle. The complete data set can be accessed in Supplementary Table S3.

The genetic control of the flavonoid pathway is well known, but the mechanisms of seasonal influence in the double cropping system are poorly understood. Therefore, one of the goals in this study was to explore the molecular changes in the berries under different cropping cycles and to correlate these changes with metabolite accumulation. Berries of three selected developmental stages (E-L 35, 36, and 38) from summer and winter cropping in 2014 were chosen to perform transcriptome analyses by RNA-Seq. PC analysis for the whole normalized gene expression was performed and most three biological replicates of each sample were well-grouped (**Figure 5**). PC1 explained 25.4% of the total variance in gene expression and separated the ripening stage of winter crop from both green and veraison stages of “Riesling” sample. PC2 explained 15.7% of the total variance and clearly separated “CS” at ripening stage of winter crop from other “CS” samples, and separation between two varieties can also be distinguished on PC2 (**Figure 5**). The expression patterns of the transcripts involved in the phenylpropanoid and flavonoid pathways were depicted as FPKM values across three developmental stages in Supplementary Table S3. In addition, the influences of cropping season on the expression of flavonoid genes were expressed as the log2-fold change of the transcript abundance in winter cropping compared to summer cropping (**Figure 6**). A subset of DEGs participating in multiple branches of flavonoid metabolism was identified in two varieties.

The early phenylpropanoid pathway, acting upstream of the flavonoid pathway branch, provides the precursors of *p*-coumaroyl-CoA for subsequent phenolic biosynthesis. The winter cropping displayed a distinct and significant upregulation of most upstream genes during ripening, among which one unigene of *Vvi4CL* (*4-coumarate: CoA ligase*, VIT_206s0061g00450) was downregulated in both cultivars. Interestingly, a recent study conducted on red blotch-infected berries found 25 suppressed genes in phenylpropanoid metabolism, while this unique *Vvi4CL* was induced by the disease during berry ripening (Blanco-Ulate et al., 2017). It is noteworthy that *p*-coumaroyl-CoA is a branch point toward flavonoid and stilbene synthesis. In the present study, the transcripts encoding stilbene synthase (STS) were increased in “CS” during the ripening process, while they decreased in “Riesling” across the three developmental stages (Supplementary Table S3). The *VviSTS* unigenes were significantly modulated by the growing season, and their transcripts were distinctly induced as the berry developed from veraison to post-veraison in both cultivars, which coincided with the accumulation of stilbenes at the onset of ripening (Deluc et al., 2011). In parallel, the cropping season greatly triggered the expression of upstream genes in the flavonoid pathway, the transcripts of which showed coordinated expression patterns with genes in the phenylpropanoid pathway, ensuring a sufficient quantity of precursors for the subsequent synthesis of flavonoid compounds.

The flavonoid metabolic pathway shares a common upstream route, and then the flux is diverted to 3′-hydroxylated or 3′5′-hydroxylated flavonoids separately via the enzyme F3′H or F3′5′H. Similar with upstream genes, including two *VviF3H* (VIT_204s0023g03370, VIT_218s0001g14310), two *VviF3*′*H* (VIT_203s0063g01690, VIT_209s0002g01090), and six *VviF3*′*5*′*H* (VIT_206s0009g02810, VIT_206s0009g02830, VIT_206s0009g02840, VIT_206s0009g02970, VIT_206s0009g03000, VIT_206s0009g03010), were significantly upregulated around the veraison stage in the winter berries versus the summer berries. Of particular interest were the transcripts encoding the F3′5′H enzyme, which were mainly expressed in the red grapes of “CS.” The transcript levels of *VviF3*′*5*′*H* were extremely low in “Riesling,” suggesting that a different transcriptional regulation mechanism for *VviF3*′*5*′*H* existed in white and red varieties (Matus et al., 2017). The simultaneous upregulation of both *VviF3*′*H* and *VviF3*′*5*′*H* transcripts in winter berries compared to summer berries of “CS” also modulated the relative abundance of the different flavonoid forms. In the current study, the ratio of *VviF3*′*5*′*Hs* to *VviF3*′*Hs* levels in “CS” was significantly and distinctly higher in winter cropping berries during ripening, in parallel with their higher ratio of 3′5′-substituted to 3′-substituted flavonoids, which was presumed to control the flavonoid composition of grape berries. Similarly, a previous study also found that the temporal and variety-specific expression of *VviF3*′*H* and *VviF3*′*5*′*H* in grapes occurred in coordination with the accumulation of the respective hydroxylated metabolites (Bogs et al., 2006; Sun et al., 2016).

The downstream flux of flavonoid metabolism in grape berries included multiple branches, and the related genes showed divergent expression patterns across the three developmental stages in the two cropping cycles. Contrary to the coordinated upregulation of upstream genes, a subset of transcripts involved in the late biosynthetic pathway was significantly repressed at some stages, such as *VviDFR* (*dihydroflavonol 4-reductase*, VIT_215s0048g01010, VIT_219s0014g04980, VIT_202s0025g01260), *VviFLS* (VIT_218s0001g03430), *VviLDOX* (*leucoanthocyanin dioxygenase*, VIT_208s0105g00380), and *VviGT5* (*UDP-glucuronic acid: flavonol 3-O-glucuronosyltransferase*, VIT_211s0052g01600), whereas other transcripts, such as *VviDFR* (VIT_216s0039g02350, VIT_218s0001g12800), *VviFLS* (VIT_211s0118g00390, VIT_218s0001g03470), *VviLDOX* (VIT_202s0025g04720), and *VviLAR* (VIT_201s0011g02960, VIT_217s0000g04150), were significantly upregulated in many cases. Of the five known *VviFLS* genes, the enzyme of which commits to flavonol biosynthesis, only two transcripts of *VviFLS4* (VIT_218s0001g03470) and *VviFLS5* (VIT_218s0001g03430) were expressed, and they showed differential expression patterns in the two cropping cycles (Fujita et al., 2006). In this study, the transcript level of *VviFLS4* was extremely low, and it was only upregulated by winter cropping at E-L 38 in “CS” and at E-L 35 in “Riesling.” Genes encoding two previously characterized flavonol glycosyltransferases, VviGT5 and VviGT6 (UDP-glucose/UDP-galactose: flavonol-3-*O*-glucosyltransferase, VIT_211s0052g01630), were co-expressed with *VviFLS4*, which is in agreement with a previous report (Malacarne et al., 2015). Czemmel et al. (2017) also demonstrated positive correlation of *VviMYBF1* with novel genes of the flavonol pathway, *VviGT3* (*flavonol glycosyltransferase*, VIT_211s0052g01580) and *VviRhaT1* (*flavonol rhamnosyltransferase*, VIT_200s0218g00170), during berry development. In the present study, the transcript of the former was detected at low levels (FPKM < 0.1), while the latter was expressed with a FPKM ranging from 5 to 15 (Supplementary Table S3). Additionally, *VviRhaT1* was significantly upregulated at E-L 35 in “CS,” which might also account for the higher flavonol in winter grapes. LAR and ANR are key regulators of flavan-3-ol and proanthocyanidin biosynthesis. The expression of *VviANR* (VIT_200s0361g00040) was higher in winter grapes at E-L 35, but no significant difference was found. Winter cropping significantly upregulated *VviLAR1* (VIT_201s0011g02960) at E-L 36 and *VviLAR2* (VIT_217s0000g04150) at E-L 38 in “CS,” while in “Riesling,” only *VviLAR2* was significantly affected, with an upregulation at E-L 36 and a downregulation at E-L 38. Comparing the expression patterns between the two seasons, *VviLAR2* in “Riesling” kept a moderate and stable level in summer cropping, while the transcript in winter cropping increased to twofold higher at veraison, and then it dropped sharply at harvest. Hence, it may be speculated that the transcript of *VviLAR2* leads to the peak flavan-3-ol content at E-L 35 in winter grapes. Furthermore, there is a range of anthocyanin biosynthetic enzymes relevant to the glycosylation, methylation, and acylation events of anthocyanin, such as UFGT (Boss et al., 1996), anthocyanin *O*-methyltransferase (AOMT; Fournier-Level et al., 2011), and anthocyanin acyltransferase (3AT; Rinaldo et al., 2015). The expression patterns of these genes involved in anthocyanin modification across three developmental stages were similar with the expression patterns of the upstream metabolic genes, the transcripts of which peaked at E-L 36, with higher levels in the winter cropping than in the summer cropping. The expression of *VviUFGT*, catalyzing the formation of anthocyanin-3-*O*-glucosides, is critical for berry coloration (Boss et al., 1996). The constantly higher expression of *VviUFGT* in winter cropping correlated remarkably well with the greater abundance of anthocyanins in winter grapes than summer grapes. The two AOMTs responsible for anthocyanin methylation (Fournier-Level et al., 2011) were also significantly upregulated in winter cropping, which failed to explain the methylation variation, since the proportion of malvidin was dramatically decreased in winter grapes. It was noticed that two transcripts, VIT_205s0062g00300 and VIT_205s0062g00310, exhibited similar patterns in the two cultivars, but they differed in their magnitude of abundances; these transcripts might encode a UDP-glucose: anthocyanidin 5,3-*O*-glucosyltransferase (53GT) with homology to a flavonol glucosyltransferase-like protein. The winter cropping significantly downregulated the expression of *Vvi53GT* in “Riesling” at E-L 31, and then upregulated it at E-L 38. In fact, the real role of VIT_205s0062g00300 and VIT_205s0062g00310 in grapes still needs further research, and the reason for their high abundances in “Riesling” is unclear.

### Transcriptional Modulation of Flavonoid Biosynthesis

Two classes of genes are required for flavonoid biosynthesis in grapes, the first class is structural genes that encode enzymes in the metabolic pathway, and the second class includes regulatory genes that control the transcription of these biosynthetic genes. The flavonoid pathway genes are known to be coordinately controlled by the interactions of R2R3–MYB, basic helix-loop-helix (bHLH), and WD40-repeat transcription factors (TFs) in response to developmental cues or external stress factors, with MYB being central to the transcriptional complexes (Heppel et al., 2013). The ternary complex of MYB/bHLH/WD40 binds to responsive elements in the promoters of biosynthesis genes, activating transcription of genes in the pathway. While many structural genes were significantly upregulated in winter cropping berries, many previously identified regulatory genes were either weakly affected or were not affected by cropping cycle (Supplementary Table S3).

VviMYBF1 regulates a narrow set of genes involved in flavonol biosynthesis, potentially comprising the genes *VviCHS* (chalcone synthase) and *VviFLS4* (also named *VviFLS1*; Czemmel et al., 2009, 2017). The expression of *VviMYBF1* was very low in both varieties, and its transcript abundance decreased across the three developmental stages, similar to a previously reported expression pattern in developing Shiraz berries (Czemmel et al., 2009). The present study showed an upregulation of *VviMYBF1* in the winter cropping cycle at E-L 38/E-L 35 in “CS”/“Riesling” (**Figure 6**), that was not well correlated with the expression pattern of *VviFLS4*. With respect to the regulation of proanthocyanidin-specific biosynthesis, many regulators have been recently characterized, including VviMYBPA1, VviMYBPA2, and VviMybPAR (Bogs et al., 2007; Terrier et al., 2009; Koyama et al., 2014). In addition, VviMYB5a and VviMYB5b, as well as the negative repressors VviMYBC2-L1 and VviMYBC2-L3, regulate the genes involved in proanthocyanidin biosynthesis and several steps in the upstream pathway (Deluc et al., 2006, 2008; Cavallini et al., 2015). The genes encoding the above TFs were expressed at some stage during ripening, and some of them showed similar expression patterns between the two varieties. It has been reported that MYB5a and MYB5b tightly exert their regulation in a temporal way during berry development, with MYB5a predominantly acting in the early stages and MYB5b acting near the later ripening process (Deluc et al., 2008; Matus, 2016). Similarly, the transcript profiles of *VviMYB5a* and *VviMYB5b* were distinct during ripening, with peak levels of *VviMYB5a* at E-L 35 and peak levels of *VviMYB5b* at E-L 38. The transcript levels of *VviMYBPA2*, *VviMYBPAR*, and *VviMYBC2-L2*, on the other hand, remained low throughout berry development, and they were not differentially expressed between the two cropping cycles. The expression of *VviMYBPA1* was significantly downregulated in winter cropping berries at E-L 35 and E-L 38 in both varieties, which correlated well with the expression of two *VviDFR* transcripts (VIT_219s0014g04980, VIT_202s0025g01260). Interestingly, the expression of two negative regulators, VviMYBC2-L1 and VviMYBC2-L3, was significantly upregulated at E-L 36 by winter cropping in “CS”; VviMYB4a was significantly upregulated as well, and it might negatively regulate flavonoid or phenolic acid synthesis (Cavallini et al., 2015). MYB14 and MYB15 were two TFs demonstrated to specifically activate STS genes (Höll et al., 2013). The expressions of *VviMYB14* and *VviMYB15* matched well with STSs profiles in “CS” rather than in “Riesling,” suggesting different transcriptional regulation of stilbene biosynthesis in two varieties.

In *V. vinifera* grapes, two *VviMYBA* genes in a single gene cluster are responsible for berry color variation and anthocyanin accumulation, among which are the *VviMYBA1* and *VviMYBA2* genes encoding putative regulators of anthocyanin biosynthesis in red grapes (but these genes are non-functional in white grapes), while *VviMYBA3* is only statistically associated with berry color without functional validation (Walker et al., 2007; Fournier-Level et al., 2009). These three *VviMYBAs* showed similar expression patterns in “CS,” namely, they were significantly upregulated by winter cropping, especially at E-L 35 and E-L 36, in parallel with the expression profiles of *VviUFGT*, *VviAOMT*, and *Vvi3AT*, and the evolution of anthocyanin levels. More recently, two bHLH proteins of VviMYC1 (VIT_207s0104g00090) and VviMYCA1 (VIT_215s0046g02560) were demonstrated to promote anthocyanin accumulation in cooperative interaction with VviMYBA1 (Matus et al., 2010; Hichri et al., 2011), the transcripts of which showed no significant variation between the two cropping cycles. *VviWDR1* (VIT_216s0098g00870), which contributed positively to the accumulation of anthocyanins, was downregulated by winter cropping at E-L 38, contrary to the expression change of *VviMYBA1*.

In fact, apart from the widely acknowledged MYB, bHLH, and WD40 TFs, some regulators that belong to the WRKY, AP2/ERF, MADS-box, GATA, and bZIP families were also positively or negatively involved in flavonoid metabolisms (reviewed by Hichri et al., 2011). A recently identified TF of VvibZIPC22 is involved in the regulation of flavonoid biosynthesis, the expression of which was induced by UV light, paralleled by accumulation of the *VviFLS4* transcript and flavonol compounds (Malacarne et al., 2016). In the present research, *VvibZIPC22* was significantly upregulated by winter cropping in both varieties, in good agreement with the accumulation of flavonols. However, no correlation was found between the expression profiles of *VviFLS4* and *VvibZIPC22*. It is relevant to mention the photomorphogenic factors from the bZIP family, which play important roles in mediating light-dependent flavonoid regulation, especially the biosynthesis of flavonols (Loyola et al., 2016; Matus, 2016). Many components in the UV-B signaling pathway that specifically perceive UV-B radiation have been identified in *V. vinifera*, including UV-B RECEPTOR 1 (UVR1, VIT_207s0031g02560), two ELONGATED HYPOCOTYL 5 grape homologs (HY5, VIT_204s0008g05210; HYH, VIT_205s0020g01090), and two CONSTITUTIVE PHOTOMORPHOGENIC 1 (COP1-1, VIT_212s0059g01420; COP1-2, VIT_210s0523g00030), which could mediate flavonol biosynthesis in grapes under UV-B exposure (Carbonell-Bejerano et al., 2014; Liu et al., 2015; Loyola et al., 2016). In southern China, UV-B radiation was shown to be higher in the summer, due to stronger sunlight and a longer duration of sunlight, although we did not detect the precise daily dose of UV-B. Concomitantly, most genes involved in the UV-B response pathway were significantly repressed in the winter cropping, except for *VviCOP1-1* (Supplementary Table S3), which aligns with the climatic conditions. Solar UV radiation was shown to enhance flavonol accumulation (Carbonell-Bejerano et al., 2014; Loyola et al., 2016). However, the changes in *VviUVR1*, *VviHY5*, and *VviHYH* expression could not match the flavonol variation between the two cropping cycles, which might be resulted from temperature-responsive changes in HY5 levels, since HY5 protein has been shown to degrade at high temperature (Kim et al., 2017; Park et al., 2017).

### Seasonal Response of Plant Hormone-Related Genes

**FIGURE 7:**
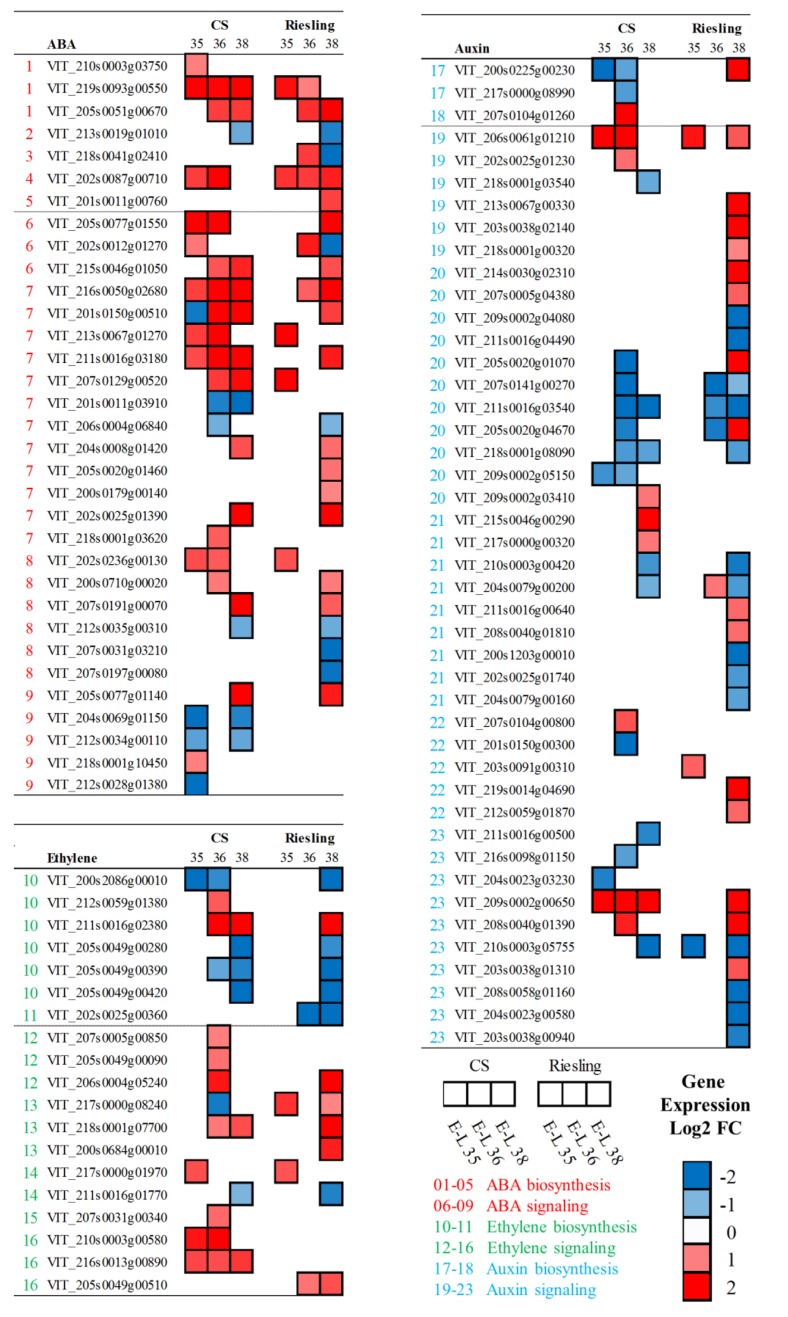
Expression profiles of DEGs related to hormone biosynthesis and signaling pathway during grape development in “CS” and “Riesling” in 2014. The log2-transformed FPKM values (Winter/Summer) are represented by the color map. Blue and red boxes indicate downregulated and upregulated transcripts, respectively, in winter berries versus large berries. The numbers indicate different components. The complete data set can be accessed in Supplementary Table S4.

Based on many years of cultivation experiences, grape farmers found the progression of ripening was different between the two cropping cycles. Since plant hormones play important roles in the ripening process of fruit, the expression of ABA and ethylene associated genes was analyzed. It was thought that *VviNCED* (*9-cis-epoxycarotenoid dioxygenase*) encodes the key enzyme for the bulk of ABA biosynthesis, and its expression correlated well with ABA accumulation (Sun et al., 2010; Young et al., 2012). The transcript abundances of *VviNCED2* (VIT_210s0003g03750) and *VviNCED3* (VIT_219s0093g00550) peaked at E-L 35 in both varieties, and they decreased after veraison. It was clear that the winter cropping upregulated three *VviNCEDs* in “CS,” especially *VviNCED3*, which showed massive fold-changes in all of the berry stages. In “Riesling,” *VviNCED3* was upregulated at E-L 35 while *VviNCED6* was significantly promoted at E-L 36 (**Figure 7** and Supplementary Table S4). The relative expression of genes involved in ABA catabolism, such as ABA 8′-hydroxylase (VIT_202s0087g00710), was also significantly increased in both varieties. ABA plays a crucial role in response to a variety of abiotic stresses, such as drought, salinity, and extreme temperature. Thus, the drought due to low rainfall in the winter cropping had a more pronounced influence on ABA induction. In the ABA signaling pathway, the transcript abundances of *VviPYR1/PYL/RCAR* (VIT_205s0077g01550, VIT_202s0012g01270, VIT_215s0046g01050) were significantly increased in winter cropping berries, while the expressions of *VviPP2Cs* (*protein phosphatase 2C*) were also significantly upregulated, except for two transcripts (VIT_201s0011g03910, VIT_206s0004g06840), which showed significant declines after veraison. For *VviSnRK2s* (*sucrose-non-fermenting1-related kinase 2*), both upregulated and downregulated transcripts were identified between two cropping cycles. ABA responsive element binding factors play a crucial role in ABA-dependent gene activation. There were no significant changes in the gene expression of *ABFs* in “Riesling,” while significant downregulation was observed in “CS,” except for *VviABF2* (VIT_218s0001g10450), which was confirmed to modulate ABA-dependent berry ripening processes, such as the promotion of phenolic synthesis and cell wall softening (Nicolas et al., 2014).

Considering the important role of ethylene in the ripening process and its cross-talk with ABA (Sun et al., 2010; Cramer et al., 2014), the expression changes in the ethylene biosynthesis and signaling pathways were also analyzed (Supplementary Table S4). Similar to the role of NCED in ABA biosynthesis, 1-aminocyclopropane 1-carboxylate oxidase (ACO) is known to determine the production of ethylene. The transcript accumulation of *VviACO1* was suggested to match the occurrence of the ethylene peak in “CS” clusters (Chervin et al., 2004). The study here also showed decreasing expression levels of *VviACO1* (VIT_200s2086g00010) as berry ripening progressed from veraison, except for a sudden rise in the summer grapes of “Riesling” at harvest. The expression of putative *VviACO1* and three other candidate genes (VIT_205s0049g00280, VIT_205s0049g00390, VIT_205s0049g00420) were significantly repressed in winter grapes in both varieties in comparison to their expression in the summer cropping. The *VviACO2* gene was expressed at high levels throughout berry development, with a significant upregulation in the winter berries of “CS” at post-veraison. Only one transcript of *VviACS* (*1-aminocyclopropane 1-carboxylate synthase*, VIT_202s0025g00360) was differentially expressed between the two cropping cycles, which peaked at E-L 35 and had higher levels in “Riesling” berries. Interestingly, winter cropping repressed the ethylene biosynthesis pathway, while the ethylene signaling pathway was greatly induced. Particularly, many genes involved in ethylene signaling, such as the ethylene receptors ethylene response 2 (ETR2) and ethylene insensitive 4 (EIN4), the negative regulators constitutive triple response 1 (CTR1), EIN3-binding F-box 1 and 2 (EBF1/2), and ethylene response factor (ERF), were significantly upregulated in winter cropping at different stages in both varieties.

Auxin plays positive roles in plant growth and delays ripening-associated processes. In grape berries, auxin is produced through the combined action of tryptophan aminotransferase related (TAR) and YUCCA (YUC) proteins in a two-step biosynthesis pathway (Böttcher et al., 2013). As expected, many transcripts in the auxin biosynthesis and signaling pathways were expressed in developing berries, peaking at E-L 35. Gene expression of the putative auxin biosynthesis members from the TAR and YUC families was analyzed. Four members of *VviTAR* were downregulated in “CS,” but only *VviTAR1* and *VviTAR2* were differentially expressed. Comparing the three developmental stages, the transcript of *VviTAR1* peaked at E-L 36 in summer cropping, while it increased gradually until E-L 38 in winter cropping. Both varieties showed similar expression patterns, but the winter cropping downregulated *VviTAR1* in “CS” while it upregulated the transcript in “Riesling.” The auxin-influx transporter AUX1 mediates the uptake of auxin, and many transcripts encoding AUX1 were upregulated by winter cropping. Auxin/indole acetic acid (AUX/IAA) proteins are a family of transcriptional repressors that play a central role in auxin response. Most *VviAUX/IAA* genes were downregulated at E-L 36 in “CS” while in “Riesling,” the effects mainly occurred at E-L 38. In addition, there were many transcripts that also showed upregulation at E-L 38 in “Riesling,” including *VviTAR1* and *VviAUX1*. Gretchen Hagen 3 (GH3) plays a role in the conjugation of indole-3-acetic acid to aspartate at the onset of grape berry ripening (Böttcher et al., 2011); one putative transcript of VIT_203s0091g00310 was upregulated at E-L 35 in winter versus summer grapes, and another of VIT_219s0014g04690 was promoted at E-L 38. The SMALL AUXIN UP RNA (SAURs) act as positive or negative regulators of auxin synthesis, and their transcription depended on the level of active auxin (Hagen and Guilfoyle, 2002). Downregulation was found to dominate the differential expression of *VviSAURs* in winter versus summer berries. Among the 10 *VviSAURs*, two (VIT_209s0002g00650, VIT_208s0040g01390) showed consecutive upregulation along three stages in “CS,” but in “Riesling,” the upregulation only occurred at E-L 38.

## Discussion

The metabolism of flavonoids in grapevine is closely correlated with environmental stresses, variety and tissue, developmental cues, and phytohormone regulation. There have been many transcriptomic studies showing the dynamics of gene expression and flavonoid accumulation in response to internal and external stimuli (Degu et al., 2014; Carbonell-Bejerano et al., 2014; Li et al., 2014; Savoi et al., 2016; Blanco-Ulate et al., 2017; Lecourieux et al., 2017; Sun et al., 2017). Here, a comparative parallel analysis was conducted on grapes grown under a double cropping system in two cultivars. To the best of our knowledge, this is the first systemic research of double cropping berries from both transcriptomic and metabolic views during the course of berry development, which enables a comprehensive description of the cropping cycle controlling flavonoid synthesis in grape during ripening.

### Winter Cropping Promotes Phenylpropanoid–Flavonoid Metabolism during Berry Ripening

Considering that the environmental conditions vary greatly in the two growing seasons, we propose that the climate factors of temperature, light, and rainfall exert great contributions to the variation of flavonoid compounds between the two cropping cycles. Investigations into the influences of climate factors on flavonoid biosynthesis in a vineyard have various approaches at different stages (reviewed by Kuhn et al., 2014). These cultural practices or environmental factors resulted in a range of differences in the levels and profiles of flavonoid compounds in grape berries. The effects of the cropping cycle on flavonoid accumulation are the result of a combination of many climate factors across the whole life cycle in the same vineyard. Notably, these three classes of flavonoids: anthocyanins, flavonols, and flavan-3-ols, respond differently to the climatic stimulus.

In grapes, flavonols are important ultraviolet light protectants and play pivotal roles in fresh fruit and the resulting wine. Flavonol accumulated in berries across the entire berry development process, showing an increase from pre-veraison to harvest in cv. “CS” but remaining relatively constant during ripening in “Riesling,” which resulted in a different content and profile of flavonol products between the two cropping cycles. It has been reported that flavonols are the flavonoids that are most drastically affected by different light levels, correlating with the expression pattern of *VviFLS4* but not with that of *VviFLS5* and coinciding with its putative transcriptional regulator, *VviMYBF1* (Czemmel et al., 2009; Matus et al., 2009; Carbonell-Bejerano et al., 2014). Contrarily, the berries in the summer cropping, which had abundant sunshine hours and high illuminance, accumulated lower amounts of flavonols than the winter grapes in both varieties. What was more interesting was the expression profiles of *VviFLS4* and *VviMYBF1*; the former was upregulated at the harvest stage of “CS” versus post-veraison of “Riesling,” while the latter was upregulated in “CS” but downregulated in “Riesling,” and both were expressed at low transcript levels. The *VviFLS5* gene was expressed at a level several times higher than *VviFLS4* and showed a downregulation in winter cropping in both cultivars. The fact that there was discrepancy in flavonol biosynthesis and the expression profiles of *VviFLS* and *VviMYBF1* in the present study indicated the involvement of post-transcriptional control or other regulators, which require further research. Notably, some differences not correlating well could be due to the fact that both shaded and sunny berries were sampled together. Besides, the deseeded berries were used to extract RNA while the flavonols were isolated from the skin, which also had an effect. Several studies have shown that light radiation plays a profound effect on flavonol synthesis, while temperature has no effect or a weak effect on their content (Spayd et al., 2002; Cohen et al., 2012; Azuma et al., 2015). Thus, the induction of flavonols in response to climatic stresses is a complex process, and the promotion by some factors in the winter cropping was suggested to have a greater effect than the offset from lower light radiation. Downey et al. (2003) displayed the two distinct periods of flavonol synthesis; the first occurred around flowering and the second occurred during berry ripening, which all coincided with the expression of *VviFLS4*. Therefore, the green berries in the winter cropping accumulated more FLS around fruit-set in response to strong light, which might have also impacted the veraison stage, contributing to larger amounts of flavonol. Additionally, the active upstream pathway in winter cropping could provide a greater abundance of precursors for the production of flavonols in grape skin.

UV-B radiation is a key environmental signal, exerting a strong effect on flavonol synthesis in grape berries. Briefly, UV-B is specifically perceived by UVR8, which in turn, function together with a positive COP1 to activate a range of signaling cascades mediated by HY5 and HYH; then, they finally regulate transcription of target genes, resulting in downstream responses (Loyola et al., 2016; Matus, 2016). Flavonol biosynthesis in grapes is suggested to be predominantly stimulated by the UV-B response pathway through the activation of HY5 and HYH (Liu et al., 2015; Czemmel et al., 2017). In addition, VviMYBF1 could act on the UV-B signaling cascade by activating HYH (Czemmel et al., 2017), thus inducing the direct and indirect activation of *VviFLS4* and several targets in the phenylpropanoid pathway, such as *VviCHS3* and *VviGT5* (Carbonell-Bejerano et al., 2014; Loyola et al., 2016). The expression pattern of the UV-B response machinery in the two cropping cycles was correlated with light radiation, but it failed to explain the variations in flavonol content in the current study, since enhanced levels of flavonols normally coincide with the upregulation of genes in the light-signaling pathway (Loyola et al., 2016).

Flavan-3-ols are monomeric subunits of condensed tannins in grape skin and seeds, contributing greatly to the body and mouthfeel of wines being produced. However, relatively little is known about the mechanism of environmental impact on flavan-3-ol production in grape skins, despite the fact that it shares common upstream steps with flavonol and anthocyanin. The grape skin had high levels of flavan-3-ols compared with other flavonoids; they accumulated from fruit-set until veraison and then declined afterward, in parallel with the expression patterns of *VviANR* and *VviLAR* (Bogs et al., 2005). In the present study, the content of total flavan-3-ol in skin was determined in two consecutive years, but it varied only in 2014. The evolution pattern of total flavan-3-ols in grape skins also varied in two cropping cycles. Since the massive production of flavan-3-ols occurred at the green stage, it was hard to correlate the gene expression profiles with its accumulation, as we only conducted RNA-Seq from the onset of ripening. However, a higher expression of *VviLAR1* or *VviLAR2* might explain the higher levels of flavan-3-ols in the winter cropping. To date, several regulatory TFs related to flavan-3-ol biosynthesis have been found in grape: the MYB positive regulators of VviMYBPAR and VviMYBPA1/PA2 and the MYB C2 repressors of VviMYBC2-L1/L3 (Bogs et al., 2007; Terrier et al., 2009; Koyama et al., 2014; Cavallini et al., 2015), many members of which also worked beyond the phenylpropanoid pathway in addition to VviMYB5a/5b (Deluc et al., 2006, 2008). According to the regulatory mechanisms of flavonoid biosynthesis in grapes, VviMYBPAR, VviMYBPA1, VviMYBPA2, and VviMYB5a are particularly involved in regulation before the onset of ripening, while VviMYB5b also controlled the general flavonoid pathway after veraison (Deluc et al., 2008; Matus, 2016). However, VviMYB5b is more related to anthocyanins in the ripening stage rather than proanthocyanidins (Cavallini et al., 2014). Thus, it was hard to correlate the flavanol evolution pattern with gene expression profiles during ripening. Additionally, Carbonell-Bejerano et al. (2013) also found that elevated temperature promoted the expression of *VviMYBPA1*, but repressed *VviMYB5a*, indicating that these regulators responded differently to external stimuli during grape development and that the regulatory network was tightly organized in a spatial and temporal way.

As specific metabolites of the flavonoid pathway, anthocyanins are synthesized only in red grapes under the strict control of multiple regulatory factors. A rapid accumulation of anthocyanins in red varieties begins from veraison until harvest, leading to more assimilated carbon flux into the production of anthocyanins rather than flavan-3-ols. The positive regulators VviMYBA1 and VviMYBA2 are specific to controlling the biosynthesis of anthocyanin in red *V. vinifera* grapes mediated through VviUFGT, while in white grapes, a retrotransposon of Gret1 inserted in the promoter region of the *VviMYBA1* gene and two non-synonymous mutations that occurred in the *VviMYBA2* coding region disrupt their regulatory function (Boss et al., 1996; Kobayashi et al., 2004; Walker et al., 2007). In the winter cropping of “CS,” both *VviMYBA1* (fold change > 1.5) and *VviMYBA2* (fold change approximately 4.0) were significantly promoted at pre- and post-veraison, they then significantly upregulated the key gene of *VviUFGT* (fold change > 2.0) at post-veraison, and finally, they contributed to the drastic accumulation of anthocyanins (fold change approximately 10) during ripening. As far as we know, little research has been conducted on double cropping grapes in subtropical climates, except for two contrasting works. One also found higher flavan-3-ol and anthocyanin levels in winter berries at harvest (Xu et al., 2011), while the other one showed no difference between the two cropping cycles at maturity (Chou and Li, 2014). Differences in grape genotype, environmental conditions, and cultural practices might explain this conflicting result. Recent studies also confirmed that high temperature (35°C) strongly reduced anthocyanin synthesis and enhanced its degradation (Carbonell-Bejerano et al., 2013; Lecourieux et al., 2017). Moreover, the repressors VviMYB4a and VviMYBC2-L1/L3 were significantly upregulated in the winter cropping at post-veraison, in accordance with the potential roles of balancing the inductive effects of activators (Cavallini et al., 2015).

Here, it is worth discussing the competition between F3′H and F3′5′H in grapes, since the profiles of flavonoids varied significantly in the two cropping cycles, especially for anthocyanins and flavonols. In contrast to the high expression of *VviF3*′*H* and *VviF3*′*5*′*H* in red grapes, the expression of the *VviF3*′*5*′*H* transcript in white grapes during ripening was extremely low, suggesting a different regulatory mechanism (Bogs et al., 2006). Thus, it was interesting to note an accumulation of the 3′5′-substituted compounds of flavan-3-ol (i.e., epigallocatechin), but not that of flavonol (myricetin), in the white “Riesling” grapes. Considering the great “loss” of the F3′5′H pathway in “Riesling” during ripening, we only discussed the competitive relation of F3′H and F3′5′H in “CS.” The expression patterns of *VviF3*′*H* and *VviF3*′*5*′*H* coordinated with the upstream pathway genes, peaking around post-veraison, but varying in magnitude. Concurrent with higher the expression ratio of *VviF3*′*H* to *VviF3*′*5*′*H* in summer grapes, there was an increase in the proportion of 3′-substituted flavonoids in the summer cropping, which is similar to a previous result induced by water deficits (Castellarin et al., 2007). Clearly, the prevalence of *VviF3*′*5*′*H* over *VviF3*′*H* would lead to more dihydromyricetin, the precursor of myricetin, epigallocatechin, and delphinidin, and in contrast, it would yield less dihydroquercetin, the precursor of quercetin, catechin, and cyanidin (Kuhn et al., 2014). The flavonol composition was almost all quercetin-type compounds (95%) at the green stage, and this fraction decreased during ripening and dropped to a low level (70% in summer versus 40% in winter) at harvest, while the flavan-3-ols were almost exclusively catechin-type compounds (85%), and its proportion showed no difference regardless of the sample stage or cropping cycle (Supplementary Table S2). With respect to anthocyanin, the effects of the cropping cycle were somewhere in between. The anthocyanin profile showed no consistent difference in 2015, whereas in 2014, the percentage of cyanidin-type compounds was significantly higher in the winter cropping cycle (15% in summer versus 25% in winter). Hence, the work here confirmed that F3′H acted as an early flavonoid gene, while F3′5′H worked at later stages (Degu et al., 2014). The higher fraction of these 3′-substituted flavonoids at harvest in summer cropping resembled the metabolic profiles of the grapes under heat or sunlight exposure (Guan et al., 2014; Lecourieux et al., 2017). A recent work showed that VviMYBA1 could specifically induce *VviF3*′*5*′*H* and promote tri-hydroxylated fractions (Matus et al., 2017). Therefore, in winter cropping, the induced F3′5′H might divert more flux to the 3′5′-substituted sub-branch and might provide more precursors to synthesize anthocyanins and flavonols. However, the production of 3′5′-substituted flavan-3-ol reached a plateau in spite of a large amount of precursors, which is likely due to flavonol-specific control or the low substrate activity of LAR for leucodelphinidin.

### Winter Cropping Advances the Onset of Veraison and Accelerates Ripening Progression

In fruits, ripening is a complex event that involves major physiological and metabolic changes controlled by plant hormones, which are also signals in response to developmental and environmental cues (Kuhn et al., 2014). However, to date, the ripening mechanism of non-climacteric fruits is still poorly understood, especially with respect to the regulation of phytohormones. Since the climatic conditions varied greatly in the two cropping cycles, it inevitably affected the initiation of veraison and the progression of ripening, which was a good opportunity to learn about the ripening mechanism in grapevines.

Understanding how plants respond to environmental stimuli is crucial to improving yield and grape quality in the field. Berry ripening is affected by multiple climatic factors, of which water deficit (Castellarin et al., 2007), light exposure (Matus et al., 2009), and damping diurnal temperature range (Cohen et al., 2012) advance the onset of ripening, while high temperature (Lecourieux et al., 2017) and shading treatment (Matus et al., 2009) delay it. In the current study, the phenological phase varied greatly between the two growing seasons. Winter cropping accelerated the duration from budburst to post-veraison, therefore advancing but also prolonging the subsequent ripening process. The difference in ambient temperature accounted for most of this phenomenon. On the one hand, the optimum temperature range between 25 and 30°C was associated with higher rates of plant growth (Buttrose, 1969; Cohen et al., 2008), and this was the case for processes from bud-break to flowering in the winter cycle. On the other hand, with respect to grape berries, low temperature and damping diurnal temperature fluctuation in the winter cropping hastened their development and ripening processes (Kliewer and Torres, 1972; Cohen et al., 2008), while the extremely high temperature in the summer might inhibit fruit growth and berry ripening. The photoperiod or the daylength is also considered a fundamental environmental signal that affects phenological development (Sreekantan et al., 2010). The phases of flowering, onset of ripening, bud dormancy, and leaf senescence are modulated by day length, together with other stress factors (Keller, 2010). This seasonal decrease in the photoperiod and temperature might also be associated with the temporal variation in ripening progression. Moreover, berry weight was negatively correlated with the onset of veraison, since the larger berry seemed to be a dilution of the smaller one, needing more time to achieve the accumulation of the primary and secondary metabolites. The reason for the advancement of veraison in wintering cropping might be, at least in part, due to the smaller berry size. More importantly, the berries in winter cropping showed a hastened increase in TSS, especially before or around veraison, and then the rate dropped to a similar level to summer berries during ripening. Another consequence of high temperature is a hastened decline of acidity in berries (Carbonell-Bejerano et al., 2013). The sugar:acid balance at harvest is an important trait of fruit quality, and a gradual fall in acidity is also coupled with a sugar increase. During the ripening process in winter, the temperature was extremely low (approximately 10°C), so it took a long time to complete the degradation of acid, resulting in more sugar accumulation, and this was the reason why winter cropping prolonged the ripening duration. Thus, the longer duration of ripening stage in winter versus summer cropping was due to the purposes to harvest high-quality grapes.

The regulation of grape development and ripening in response to external or internal cues involves a dynamic interplay among hormones. Albeit unclearly, the functional reciprocity among plant hormones was suggested to control ripening transitions and progression (Sun et al., 2010; Böttcher et al., 2013; Pilati et al., 2017). The transition of ripening, termed as veraison, is accompanied by the modulation of hormones, concurrently with many physical, chemical, and physiological changes (Castellarin et al., 2016). Here, most genes encoding NCED/ACO/TAR, the key enzymes of ABA/ethylene/auxin biosynthesis, showed coordinated expression patterns, peaking around pre-veraison, in spite of their different expression levels. Particularly, during early berry development, the level of endogenous auxin was high and peaked around veraison, then its rapid decline occurred from pre-veraison, followed by sequential increases in ethylene and ABA content (Böttcher et al., 2013; Kuhn et al., 2014). Several studies confirmed the ethylene peak preceded the ABA peak at pre-veraison, and they suggested the trace ethylene could induce the expression of *VviNCED* and therefore the biosynthesis of ABA (Chervin et al., 2004; Sun et al., 2010). In turn, the exogenous ABA treatment on pre-veraison berries also triggered ethylene biosynthesis, suggesting a functional and positive interaction between ABA and ethylene (Sun et al., 2010; Pilati et al., 2017). In the present study, genes in ABA-related pathway were significantly upregulated in winter cropping cycle, which might explain the advance of ripening initiation.

The applications of ABA and ethylene before veraison seemed to accelerate the onset of berry ripening, whereas the synthetic auxins treatment delayed the ripening initiation (Sun et al., 2010; Ziliotto et al., 2012). Ziliotto et al. (2012) found an “antagonistic” effect between auxin and ethylene, and a “synergistic” effect between auxins and ABA. The genes involved in ABA biosynthesis and perception were repressed by auxin spraying at pre-veraison, while the ethylene biosynthetic pathway was triggered (Ziliotto et al., 2012). Ziliotto et al. (2012) found a peak of ethylene biosynthesis genes coincided with high expression levels of auxin biosynthesis genes by a pre-veraison auxin analogs treatment, leading the berries back to the pre-veraison stage. Similarly, when the pre-veraison fruits were treated with ethylene-releasing compound, the transient increase of auxin specifically induced by ethylene would counteract the positive effect of excess auxin, thus delaying the initiation of ripening phase (Böttcher et al., 2013). In this context, *VviACO1*, the transcript that was confirmed to be consistent with the ethylene peak (Sun et al., 2010), was induced by summer cropping, together with *VviTAR* in “CS,” probably resulting from the strong antagonism between auxin and ethylene.

As regards to auxin degradation, it has been shown that GH3 determined this process by conjugation of auxin with amino acids at pre-veraison, and it positively responded to the application of exogenous ABA and ethylene, representing a signal of the berry ripening (Böttcher et al., 2010). The decrease of auxin levels after veraison was probably due to the formation of IAA-Aspartate, which was closely correlated with the *VviGH3* transcript levels (Böttcher et al., 2010; Corso et al., 2016). And GH3-1 was claimed to be responsible for the auxin homeostasis at pre-veraison (Böttcher et al., 2010). Here, the *VviGH3-1* (VIT_203s0091g00310) expression was peaked at E-L 35 and remained at high levels at E-L 36 and E-L 38, in agreement with previous report (Böttcher et al., 2010). The transcript of *VviGH3-1* was upregulated at E-L 35 in winter grapes compared to summer grapes, with a 0.5-fold higher in “CS” and a 1.2-fold higher in “Riesling” (Supplementary Table S4). This behavior of *VviGH3-1* might be associated to the earlier onset of veraison in winter cropping cycle, since the action of GH3 could potentially control of berry ripening rate (Böttcher et al., 2010, 2013; Corso et al., 2016).

Taken together, the differential expression pattern of hormone-related transcripts between the two cropping cycles explained the ripening variation at a hormone level. In general, ABA is acted as a stress-stimulated signal, and the water deficit and low temperature in winter cropping acted as a positive regulator of ABA production (Deluc et al., 2009). Furthermore, coloring by the synthesis of pigments is another major change during the onset of ripening (Castellarin et al., 2016). The deeper color with more anthocyanins in winter cropping also coincided with the transcript levels of *VviNCED*, reproducing the major effects of ABA on the rise of anthocyanins, together with a sugar increase (Wheeler et al., 2009).

### Final Remarks

Climate conditions have far-reaching implications for grape cultivation in the field. The extremely high temperature and frequent, intense precipitation in the conventional grapevine growing cycle take a major toll on grape yield and quality in southern China. Thus, the development of a double cropping system per year is a great breakthrough, not only minimizing the impact of bad weather in a subtropical monsoon climate but also improving the quality and yield of out-of-season grapes. Our research here was mainly focused on the flavonoid metabolism, since the grapes grown under two cropping cycles showed distinctly different phenolic compounds in addition to their differences in skin coloration. In addition, we demonstrated that the winter cropping cycle promoted the biosynthesis of flavonoids by (i) avoiding many types of detrimental weather events and making good use of the abundant heat and light resources in southern China; (ii) prolonging the duration of ripening stage to give the berry more time to accumulate flavonoid compounds; (iii) altering the expression patterns of flavonoid-related TFs, particularly with the upregulation of *VviMYBA1*, *VviMYBA2*, *VviMYBF1*, and *VviMYB5a* and the downregulation of *VviMYBPA1*, which in turn, greatly induced the flavonoid biosynthetic genes; (iv) triggering the ABA-related ripening processes, which also positively coincided with anthocyanin accumulation; and (v) correlating with their smaller berries and higher sugars. Thus, the alterations in ripening regulatory networks and the flavonoid biosynthetic pathway probably mainly occurred at pre-veraison, leading to a great increase in metabolic gene expression around post-veraison and subsequent flavonoid accumulation.

## Author Contributions

JW, X-JB, and C-QD designed the experiments on vineyard samples. M-MC, GC, and X-JC promoted the double cropping system and sampled the grapes. W-KC processed the samples for RNA isolation for RNA-seq analysis and flavonoid extraction for HPLC-MS/MS analysis, and drafted the manuscript. X-HY participated in the process of flavonoid extractions. R-RG, YW, and LH provided statistical and bioinformatics analysis. FH and JW revised the manuscript and provided suggestions. All authors contributed to discussion of the results and approved the final manuscript.

## Conflict of Interest Statement

The authors declare that the research was conducted in the absence of any commercial or financial relationships that could be construed as a potential conflict of interest.

## Footnotes

1 http://www.metaboanalyst.ca/faces/home.xhtml

## Supplementary Material

**FIGURE S1 |** The evolution of berry fresh weight of ‘Cabernet Sauvignon’ and ‘Riesling’ under double cropping system in 2014 and 2015.
