# Supplementary figures and images for: Dissecting the Variations of Ripening Progression and Flavonoid Metabolism in Grape Berries Grown under Double Cropping System
Source: Front Plant Sci. 2017 Nov 10;8:1912. doi: 10.3389/fpls.2017.01912 (PMC5686318; doi:10.3389/fpls.2017.01912)

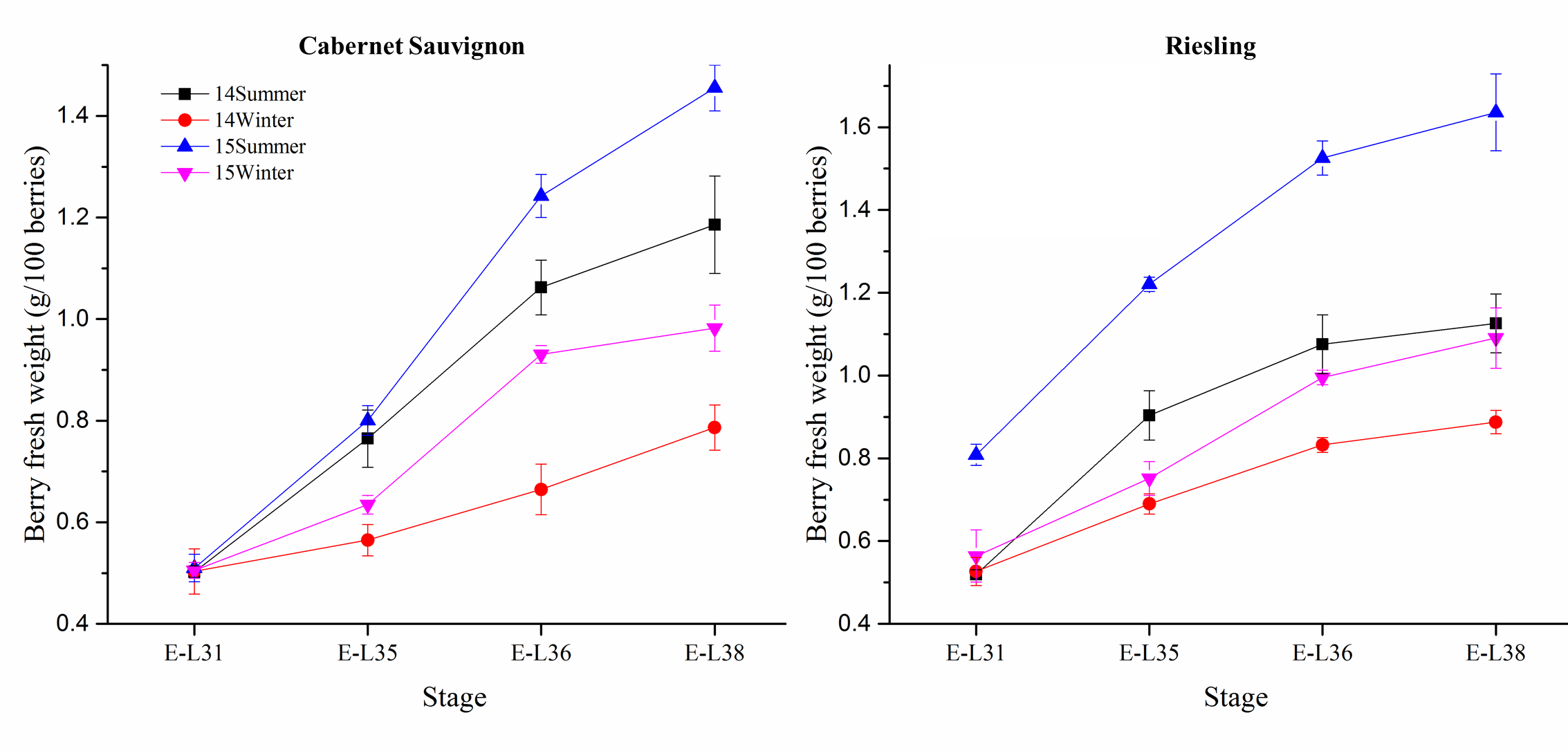

Supplement: Supplementary file 1 [file Image_1.TIF]
